# The Effect of Planning, Strategy Learning, and Working Memory Capacity on Mental Workload

**DOI:** 10.1038/s41598-020-63897-6

**Published:** 2020-04-27

**Authors:** Thea Radüntz

**Affiliations:** Federal Institute for Occupational Safety and Health, Work and Health, Mental Health and Cognitive Capacity, Berlin, 10317 Germany

**Keywords:** Problem solving, Stress and resilience

## Abstract

In our modern society, planning and problem solving are crucial for handling a wide range of situations. Investigation of the experienced mental workload connected to planning, strategy learning, and working memory capacity is of particular interest for adjusting conditions according to the mental state of the individual. In our study, we examined 21 subjects during a planning and a working memory task. We applied the method of Dual Frequency Head Maps (DFHM) from the electroencephalogram for capturing mental workload objectively. We evaluated the DFHM-workload index and performance data during the learning and main phase of the planning task and linked the results to subjects’ working memory capacity. The DFHM-workload index indicated that subjects with higher working memory capacity experienced a gradual decrease in mental workload during strategy learning of the planning task. However, the effect of learning on mental workload disappeared during the main phase.

## Introduction

Planning is a basic task in work and everyday life. In order to solve a problem, we firstly create a mental representation of the current situation and the goal state and plan the steps we need for transforming the initial state to the goal state^1^. Thereby, we generate multiple sequences of sub-goal states, rate their consequences, make decisions, and carry out actions, while continuously monitoring the outcome^2^. During planning, the working memory capacity plays an important role for maintaining and coordinating the sub-goal sequences^2–4^. Working memory defines the ability to temporally maintain information in mind and is linked not only to planning and problem solving but also to comprehension, reasoning, and learning^5^. Furthermore, working memory load is strongly connected to the experienced mental workload^6,7^ that can be conceived as the amount of cognitive demands required for task solving related to the available cognitive resources^8–11^.

Mental workload was often linked to mental health and human performance^12–18^. Objective registration and evaluation of mental workload is particular important in order to minimize errors and increase the safety of persons. Especially in our modern society, where planning and problem solving are crucial for handling a wide range of situations, the experienced workload as connected to planning, strategy learning, and working memory capacity is of particular interest. Understanding the interrelation between these constructs may contribute to adjust conditions, facilitate learning, enhance planning, and reduce mental workload.

A number of authors studied planning using the Tower of Hanoi (TOH) task and its connection to working memory^4,19–23^ and found a connection between both^24,25^. Research on how planning and working memory relate to each other regarding their induced mental workload is rare. However, several researchers found that planning includes the interaction of working memory, inhibitory control, and cognitive flexibility and can be seen as a higher-order executive function that integrates core cognitive processes^26–28^.

The study by Schiff and Vakil^29^ investigated the connection between planning and learning. The authors employed the TOH task because they considered it to be particularly appropriate for the assessment of problem solving and learning of complex cognitive procedures. They stated that the learning phase starts with the first engagement with the task (i.e., subjects’ baseline performance) and continues with rapid improvements during repeated practice within seconds to minutes. Study’s findings emphasized a trade-off between younger and older children during the learning phase that became evident through faster speed and greater accuracy for the older ones. Schiff and Vakil^29^ argued that there exists only one further study by Beaunieux *et al*.^30^ that examined learning effects by means of the TOH. Aside from this, working memory is needed for concept formation and for controlling processes as well as remember strategies that are all important for learning^5^. Several studies suggested that learning can be facilitated by increased working memory capacity^31–37^. Thus, the relation between the amount of available cognitive resources and cognitive demands required for task solving during learning should be reflected accordingly by registration of mental workload. A study that connects and investigates these aspects is not present yet.

Research also indicated a quick saturation after a fast learning effect^29,38,39^. Specifically, after a short learning phase the performance became stable for consecutive trials within a session^38^. Despite that, the performance might continue to improve again on subsequent daily sessions^38^. The time course of learning follows a curve that gradually reaches an asymptote but after intense practice and rehearsal the learned skill could become automatic^40^. This trend of fast improvement followed by a floor effect of performance can be observed also in the figures of the TOH study by Schiff and Vakil^29^ for younger as well as older children. Human performance and mental workload were often linked to each other^17,18^ and their relation was frequently outlined by the Yerkes-Dodson curve^41,42^. Consequently, a quick saturation in performance after a fast learning effect, should be also prevalent in the registration of mental workload.

As far as we know there exists only one study related to mental workload and planning. Hardy and Wright^43^ manipulated the difficulty of the TOH task and assessed the workload using the NASA-TLX questionnaire^44^ as a subjective method for workload registration. Thereby, workload ratings increased with increasing TOH difficulty and individual performance on the TOH correlated with the subjective ratings. The authors suggested that mental workload did not only reflect task’s cognitive demands but also the cognitive abilities of the performer. That means that although subjects could reveal similar task performance, they might experience different levels of workload. Hardy and Wright^43^ stated that measuring workload during cognitive tests provided additional information about the cognitive state of the subject and captured individual differences.

However, the assessment of workload using subjective questionnaire methods has a number of drawbacks. Subjective registration of mental workload is only possible in retrospect and the questionnaire method might alter subject’s mental state by imposing additional demands. An objective and reliable method for measuring instantaneous mental workload continuously over time would be more beneficial.

Over the past 50 years, different physiological parameters (e.g., heart rate and derived parameters, electrodermal activity, body temperature, etc.) have been evaluated for their validity regarding continuous mental workload registration. In last century’s 90 s, the ability of the electroencephalogram (EEG) for registering mental workload was evaluated and served as a starting point for the use of the EEG in applied research. Basically, changes in the alpha-frequency (8–12 Hz) and theta-frequency (4–8 Hz) band powers related to mental workload have been confirmed many times. Thereby, the majority of workload studies dealt with the analysis of the EEG during cognitive tasks related to working memory and executive control^45–49^. In a review article, Borghini *et al*.^50^ provided a detailed overview of the measurement of neurophysiological signals for the determination of mental workload and confirmed essentially the known relations. In recent years, classifiers were increasingly used for the separation of workload levels. The feature vectors derived from the EEG revealed varying complexity and extent, and frequency bands were taken differently into account. The used EEG parameters were, for example, the amplitude of the EEG, spectral power of different frequency bands and different EEG channels^7,51–55^. The focus was on frontal, parietal, and occipital EEG channels according to previous findings. Independent component analysis (ICA) was used to determine specific reactions of spatio-temporal different sources^56^ and allowed the successful detection and elimination of artifacts^57–59^.

Nevertheless, different cognitive strategies in task solving, both intra- and inter-individually, can influence the classification results of mental workload. Additionally, the question arises whether machine learning algorithms provide reliable and reproducible results over time. In particular, the need for appropriate retraining of the classifier regarding subjects and tasks poses additional demands for the investigation of the interrelations between planning, strategy learning, working memory capacity, and mental workload. To the best of our knowledge, there is no other study currently available that investigated the interactions of working memory, learning effects during planning, and objective mental workload registration using the EEG.

In our prior work we developed a mental-workload classifier that does not need retraining, neither for new subjects nor for new tasks^60^. In a laboratory study conducted with 54 subjects which executed well-established cognitive tasks, we developed the so-called Dual Frequency Head Maps (DFHM). These head maps consist of personalized spectral features and their spatial occurrence (i.e., frontal theta-band and parietal alpha-band powers). Support vector machines are used for classification in three classes: low, moderate, or high workload. Under laboratory conditions, we successfully proved the DFHM method as universally applicable for mental-workload indexing.

In the current study, we applied the DFHM method for capturing mental workload objectively during a planning and a working memory task. We employed the TOH as a planning task and the automated orientation span (AOSPAN) task as a working memory task. The aim of our study was the investigation of the effect of planning, strategy learning, and working memory capacity on mental workload. In a first step, we aimed to show that a higher-level executive function like planning involving several core cognitive processes^26–28^ imposes a higher mental workload than a working memory task as it binds more cognitive resources. Next, we investigated interrelations between planning, strategy learning, working memory capacity, and mental workload according to the last two hypotheses.

1. Execution of a planning task induces higher mental workload compared to a working memory task.
2. A higher working memory capacity contributes to a better strategy learning and thus to a gradual decrease in mental workload during the learning phase of the planning task.
3. After the learning phase, the effect of strategy learning on mental workload disappears during increasing task load.

## Methods

### Procedure, Tasks, and Subjects

For our investigation we employed the TOH and AOSPAN tasks. Their implementation was realized with the E-Prime application suite. All subjects executed both tasks in counterbalanced order.

**Figure 1:**
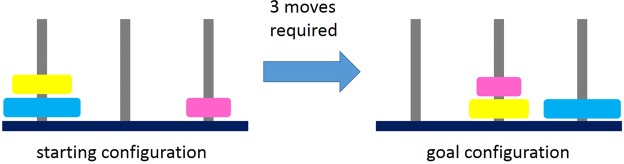
Computerized version of Tower of Hanoi. Subjects were required to transform the starting configuration into the goal configuration by three moves.

The TOH task consists of three pegs with discs of graduated size. Subjects were asked to transform the starting configuration into a given goal configuration (Fig. 1) in as few moves as possible. For this, they had to select a top disc from the source peg and place it to a destination peg. They were allowed to move only one disc at a time and they were not allowed to place big discs on smaller ones. The experiment started with a small instruction procedure where the TOH task was explained to the subjects. For familiarizing themselves with the clicking procedure during the task, subjects were asked to execute three trials with 1, 2, and 3 moves required to reach the goal configuration. Thereafter, the main experiment started including a learning phase and a main phase. The learning phase consisted of 3 trials with 3 discs each and 5, 6, and 7 moves required to reach the goal state. The main phase consisted of 3 trials with 4 discs and 7, 11, and 15 moves. In order to reach the goal-state configurations with the least-possible moves, subjects were instructed to plan their actions before starting. The number of least-required moves was given to them before each trial. If a move was not optimal and would result in a greater number of moves, they got an error message and had to start the trial again. There was no time limit set, neither for the planning time nor for task solution in general, for avoiding the tendency of a speed and accuracy trade-off. Furthermore, subjects should make full usage of the time before their first move, which was used later for performance evaluation of planning time, instead of planning during the movements.

The AOSPAN task was administered as a working memory task in the version developed by Unsworth *et al*.^61^. It was translated in German and adapted accordingly. Subjects were asked to memorize letters in the order presented while simultaneously solving math problems. The math problems required to click as soon as subjects knew the answer. After the click a number was presented and subjects had to judge if it was the right answer to the problem. Then a letter to be memorized was shown. At the end a recall slide was presented asking them to select the letters shown in the correct order. Finally, subjects got feedback about both their memory and math performance. Furthermore, the subjects were instructed to keep the percentage number indicating their math performance above 85%. The AOSPAN training took place directly before the actual task as described in Unsworth *et al*.^61^. The math practice of the task aimed to calculate for each person how long they needed to solve the math problems. Each individual’s mean (plus 2.5 SD) was used during the main AOSPAN task as a time limit for the math operations in order to account for individual differences. According to Unsworth *et al*.^61^, the time limit serves to prevent participants from rehearsing the letters when they should be solving the operations.

The participating subjects needed about 25 min to complete both tasks. Performance evaluation for the TOH task was done by analysis of individual error rates and planning time until their first move. The working memory capacity of the subjects reflected by the AOSPAN task was calculated by means of the sum of correctly recalled letters from only the sets in which all characters were recalled in correct serial order. Similar to Unsworth *et al*.^61^, we refer to it as absolute score.

We examined 21 subjects in the age between 22 and 64 years (2 female, 19 male, mean age 38 ± 11). All subjects had a background in science or engineering associate education. All of the investigations acquired were approved by the local review board of our institution and complied with the tenets of the Declaration of Helsinki. All procedures were carried out with the adequate understanding and written consent of the subjects.

### EEG and DFHM-Workload index

Biosignal processing and all calculations were done with MATLAB.

**Figure 2:**
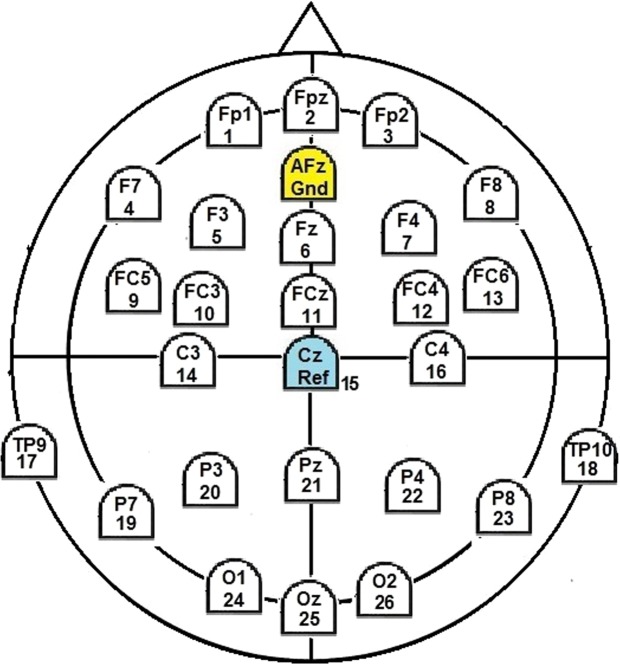
EEG layout used.

For EEG registration we used g.tec’s g.LADYbird/g.Nautilus system with 25 active electrodes placed at positions according to the 10–20 system (Fig. 2). Registration was carried out with a sample rate of 500 Hz and with reference to electrode Cz. For signal recording we used g.tec’s Matlab interface.

After recording, the EEG was filtered with a bandpass filter (order 100) between 0.5 and 40 Hz. Independent component analysis (ICA, Infomax algorithm^62^) for artifact rejection was applied to the signal. In order to increase topographical localization, we applied a simple Hjorth-style surface Laplacian filter using 8 neighbours^63^. This spatial high-pass filter was aimed to attenuate large-scale scalp signals and amplify localized signals.

The artefact-free EEG was transformed to average reference and cut into segments of 1 s length, overlapping by 0.5 s. By means of Fast Fourier Transformation (FFT) we computed the workload relevant frequency bands (theta: 4–8 Hz, alpha: 8–12 Hz) over the segments and generated the DFHM as outlined in the article by Radüntz^60^. In brief, we applied a theta-bandpass filter to the signals of the frontal electrodes and an alpha-bandpass filter to the signals of the parietal electrodes and calculated for each participant, each electrode, and each segment the z-scores of theta and alpha band power. The compilation of the z-scores of the theta band power from the frontal electrodes and alpha band power from the parietal electrodes constituted the DFHM for each EEG segment. The individual mean and standard deviation for z-score calculation were obtained from subject’s segments of the first minute of EEG recordings. These consisted not only of the two tasks relevant for this article but also of six rest measurements and eight different workplace tasks familiar to the subjects. They were conducted during a following two-day experiment and are not subject of this article.

We used the already trained SVM classifiers from the laboratory study^60^ to classify the DFHM of each subject from the tasks’ segments. Every 0.5 s we obtained a value determining if the segment belongs to low, moderate, or high workload. We applied a moving-average time window of 6 s and adjusted the result in order to gain a DFHM-workload index as percentage value between 0 (all DFHM classified as low) and 100 (all DFHM classified as high).

### Statistical analysis

For evaluating our first hypothesis confirming the expected higher mental workload during the the planning task, we calculated the DFHM-index average over the TOH and AOSPAN tasks. The Shapiro-Wilk test did not show normal distribution for the differences of the DFHM-index averages between both tasks. Thus, a Wilcoxon signed-rank test was calculated.

For investigating the effect of working memory capacity on mental workload during strategy learning of a planning task (hypothesis 2), we employed the DFHM-index averages of the three TOH trials of the learning phase. The Shapiro-Wilk test showed a normal distribution for the three DFHM-index averages. Thus, we carried out an analysis of variance (ANOVA) with the items’ mean DFHM index as dependent variable. We utilized a repeated-measures design with one within-subject factor for the number of required moves (three levels: 5, 6, and 7 moves) and one between-subject factor for the working memory capacity (two levels). The latter was calculated using the median of the absolute score of the AOSPAN task. Subjects with an absolute score below the median of 43 were classified as low working memory capacity subjects (n = 10), the remaining as subjects with high mental workload capacity (n = 11). General differences between the levels were examined and tested with a post-hoc test (Bonferroni corrected). Additionally, we evaluated subjects’ planning times and the number of errors (i.e. number of restarts) for each TOH trial. The Shapiro-Wilk test did not show normal distribution, neither for the planning time nor for the number of errors. For achieving a normal distribution for the further analysis, we computed the logarithm of the planning time. Thus, we were able to proceed in the same way as described above and conduct a repeated-measures mixed ANOVA with one within-subject and one between-subject factor. Computation of the logarithm of the number of errors did not yield normal distribution. Hence, statistical analysis of the number of errors was conducted via non-parametric Friedman test of differences among the repeated measures. Dunn-Bonferroni post-hoc tests were calculated for the examination of the differences between the levels.

Finally, we addressed the issue of mental workload related to planning after the learning phase (hypothesis 3). We employed the DFHM-index averages, planning times, and number of errors of the three TOH trials during the main phase. The Shapiro-Wilk test showed similar results for all variables as during the learning phase. We carried out two repeated-measures mixed ANOVA with one within-subject and one between-subject factor, one for the DFHM index and one for the logarithm of the planning time. A non-parametric Friedman test of differences was conducted for the number of errors among the repeated measures. Dunn-Bonferroni post-hoc tests were calculated for the examination of the differences between the levels.

Statistical calculations were conducted using SPSS and the significance threshold was set at 5%.

## Results

### Planning task causes higher mental workload than working memory task

**Figure 3:**
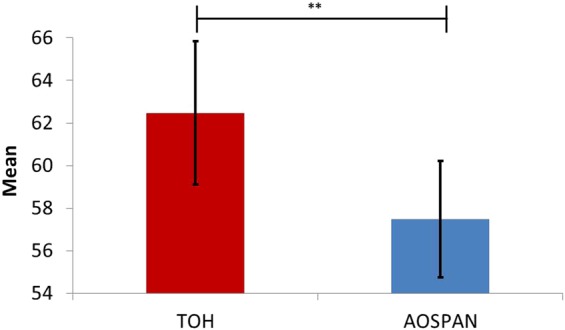
Mean DFHM-workload index during TOH and AOSPAN tasks (Wilcoxon signed-rank test differences: **0.001 < p ≤ 0.01; error bars indicate 95% confidence interval).

The Wilcoxon signed-rank test indicated significant mental workload differences between the TOH and AOSPAN tasks (T = 26, z = −3.11, p = 0.002, r = 0.48). The mental workload assessed by the DFHM-workload index from the EEG was higher for the TOH than for the AOSPAN task. Descriptive statistics are presented in Table 1 and Fig. 3 shows the results.

**Table 1:** Descriptive statistics of the dependent variables related to research hypotheses’ conditions (WM: working memory).

	DFHM-workload index	Planning time [s]	Errors
Condition	Mean ± SD, median [min, max]	Mean ± SD, median [min, max]	Mean ± SD, median [min, max]
AOSPAN, whole task^*a*^	57.5 ± 6.0, 56.6 [48.4, 67.5]	–	–
TOH, whole task^*a*^	62.5 ± 7.4, 63.6 [42.5, 73.9]	–	–
TOH learning, 5 moves			
Lower WM capacity^*b*^	63.0 ± 6.9, 64.8 [52.2, 72.0]	22.5 ± 16.2, 20.2 [5.1, 58.5]	1.2 ± 2.2, 0 [0, 7]
Higher WM capacity^*c*^	65.4 ± 10.3, 65.0 [43.8, 81.5]	20.5 ± 12.2, 17.1 [5.3, 42.5]	0.9 ± 1.2, 1 [0, 4]
TOH learning, 6 moves			
Lower WM capacity^*b*^	62.9 ± 7.8, 64.8 [47.9, 70.4]	14.1 ± 9.6, 12.1 [4.9, 37.5]	0.6 ± 1.6, 0 [0, 5]
Higher WM capacity^*c*^	61.8 ± 11.1, 62.9 [35.3, 75.5]	16.2 ± 11.8, 10.3 [5.1, 38.0]	0.5 ± 0.9, 0 [0, 3]
TOH learning, 7 moves			
Lower WM capacity^*b*^	64.9 ± 8.1, 66.4 [51.8, 77.0]	20.3 ± 13.4, 17.1 [6.9, 45.4]	1 ± 1.3, 0.5 [0, 4]
Higher WM capacity^*c*^	60.7 ± 9.0, 59.8 [48.4, 79.0]	15.7 ± 11.2, 11.8 [4.5, 36.0]	0.6 ± 0.7, 1 [0, 2]
TOH main, 7 moves			
Lower WM capacity^*b*^	63.9 ± 8.0, 66.0 [48.1, 76.6]	10.9 ± 7.8, 7.8 [4.5, 26.4]	0.2 ± 0.6, 0 [0, 2]
Higher WM capacity^*c*^	60.2 ± 10.7, 63.4 [40.0, 73.2]	15.3 ± 9.7, 10.5 [5.2, 35.0]	0.2 ± 0.4, 0 [0, 1]
TOH main, 11 moves			
Lower WM capacity^*b*^	63.4 ± 6.1, 64.3 [53.1, 70.4]	20.1 ± 17.7, 15.4 [6.8, 64.6]	1.1 ± 1.4, 1 [0, 4]
Higher WM capacity^*c*^	63.1 ± 11.4, 62.1 [37.6, 78.7]	16.5 ± 13.4, 9.0 [2.6, 38.2]	0.6 ± 0.8, 0 [0, 2]
TOH main, 15 moves			
Lower WM capacity^*b*^	64.3 ± 5.8, 65.3 [53.8, 71.4]	18.8 ± 10.4, 18.2 [8.9, 45.5]	2.6 ± 4.2, 1 [0, 13]
Higher WM capacity^*c*^	64.4 ± 10.0, 66.7 [42.7, 76.0]	19.4 ± 15.7, 12.8 [6.5, 56.3]	1 ± 1.6, 0 [0, 5]

*Note*. ^*a*^All subjects: N = 21, ^*b*^Subjects with lower WM capacity: N = 10, ^*c*^Subjects with higher WM capacity: N = 11.

### Higher working memory capacity contributes to workload decrease during strategy learning of planning

**Figure 4:**
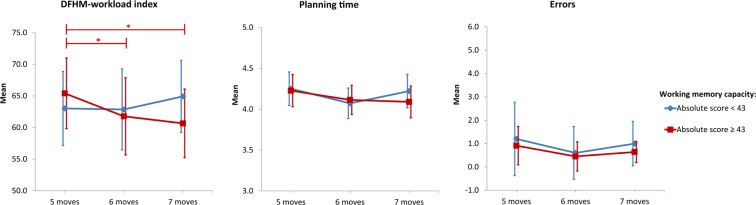
Mean values of DFHM-workload index (left), logarithm of planning time (middle), and errors (right) during the learning phase of the TOH task for subjects with lower (blue) and higher (red) working memory capacity as indicated by the absolute score of the AOSPAN task (Bonferroni corrected post-hoc tests: *0.01 < p ≤ 0.05; error bars indicate 95% confidence interval).

The mixed ANOVA yielded a significant interaction effect of requested moves and working memory capacity on mental workload (F(2, 38) = 3.62, p = 0.036, *η*^2^ = 0.159). For subjects with higher working-memory capacity the DFHM-workload decreased during the learning phase. Post-hoc analysis indicated that workload decreased significantly from the initial to the second (p = 0.031) and third trial (p = 0.025). For subjects with lower working-memory capacity, we were not able to obtain any significant workload differences during the learning phase. Evaluation of planning time and errors did not reveal any significant effects for the number of requested moves or subjects’ working memory capacity during the learning phase. Descriptive statistics are presented in Table 1 and Fig. 4 presents the results.

### Learning effect on mental workload disappears after the learning phase

**Figure 5:**
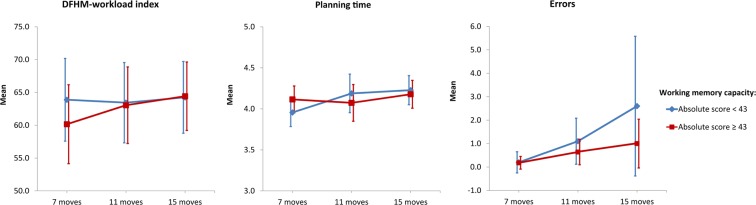
Mean values of DFHM-workload index (left), logarithm of planning time (middle), and errors (right) during the main phase of the TOH task for subjects with lower (blue) and higher (red) working memory capacity as indicated by the absolute score of the AOSPAN task (error bars indicate 95% confidence interval).

During the main phase, no significant learning effect could be obtained. This applied for mental workload as well as for planning time where mixed ANOVA calculations showed no significant effects of the number of requested moves or subjects’ working memory capacity. The non-parametric Friedman test revealed a general significant change in the number of errors for the lower working memory capacity subjects (*χ*^2^ = 8.960, df = 2, n = 10, p < 0.011). Nevertheless, subsequently conducted post-hoc tests did not reveal significant differences between the levels. For the higher working memory capacity subjects this effect was not prominent at all. Descriptive statistics are presented in Table 1 and Fig. 5 illustrates the results.

## Discussion

In our study, we investigated the effect of planning, strategy learning, and working memory capacity on mental workload. For assessing mental workload, we used the DFHM method that was previously developed in a laboratory setting and is based on the EEG. In the current study, 21 subjects participated and completed the TOH and AOSPAN tasks in randomized order. We registered the EEG and computed the DFHM-workload index for each subject and task. We did not retrain the classifiers neither for the new tasks nor for the new subjects.

The DFHM-workload index was significantly higher for the TOH than for AOSPAN task as stated by hypothesis 1. This indicated that planning imposed higher mental workload suggesting that more cognitive resources were required during planning than working memory task. The result was consistent with literature that stated that planning is a higher-order executive function that integrates core cognitive processes such as working memory, inhibitory control, and cognitive flexibility^26–28^. Although attentive readers could argue that the time limit set for the math operations during the AOSPAN task might result in time pressure and increase mental workload, our results did not support this assumption.

More insight regarding intra-individual differences linked to strategy learning and mental workload during planning was gained from subject clustering by means of the absolute score from the AOSPAN task as an indicator for subjects’ working memory capacity. During the learning phase of the TOH task, we were able to obtain a significant interaction effect between task load and working memory capacity on mental workload. Thereby, mental workload of subjects with higher working memory capacity significantly decreased while the workload of subjects with lower working memory capacity did not yield significant changes. The effect was particularly prominent for the mental workload assessed by the EEG whereas the number of errors and planning time showed only a weak tendency in that direction. This fits well the assumption by Hardy and Wright^43^ that mental workload reflects the cognitive abilities of the performer, captures individual differences, and reveals additional information about the cognitive state although task performance might be similar. We concluded that a higher working memory capacity contributes to workload decrease during strategy learning of planning as suggested by hypothesis 2. Nevertheless, learning is traditionally associated with a change in behavior^64^ and one could ask if a reduction in mental workload can indicate a learning process when there is no such change. According to the definitions of different authors^8–11^, mental workload reflects the amount of cognitive resources required for task solving. In our experiments, subjects with higher working memory capacity needed less cognitive resources for maintaining their performance although the number of required moves gradually increased during the learning phase. Consequently, we suggested that this result indicated an initial learning process on neurological level that might produce behavioral changes after longer practice. Considering the obtained tendency of performance enhancement, this assumption seems rational. However, further studies should allow subjects to perform the same version of the task more times for providing statistical-significant evidence. A possible explanation that performance changes did not reach the significance level might be also related to the higher educational background of our subjects. This might have impacted the performance by a floor effect as well. Finally, we want to call attention to a study by Huang *et al*.^65^ with supporting results for our assumption. The research was concerned with driving learning. The authors found that later stages of motor learning increased metabolic efficiency but did not reveal any gains in performance.

As task load of the planning task increased during the main phase of the TOH, the learning effect disappeared and mental workload increased regardless of subjects’ working memory capacity. The DFHM-workload index of both subject clusters converged at the most demanding trial. Conforming to hypothesis 3 results indicated a quick saturation after the short learning phase. This was particularly true for subjects with higher working memory capacity that have previously experienced a fast learning effect. Even though we were able to detect a tendency to more errors for the subjects with lower working memory capacity, the pairwise comparisons between the levels did not become significant for none of our variables. The subjects with lower working memory capacity did not seem to have learned the task at all, since at no point did the DFHM-workload index display refinement nor did performance improve. In addition, in the main phase of the experiment, the performance of the low working memory capacity group tended to reduce with no apparent change in workload. In other words, although subjects invested the same amount of cognitive resources their performance got worst with increasing task difficulty. All facts together support our previous suggestion that mental workload indicates an initial learning process on neurological level that may result in behavioral changes during the main practice.

A limitation of our study was our small sample set. Future studies should involve more females, subjects with different educational levels, and also older participants. In our study, the educational background of our subjects was in science or engineering and equally high among them. Affinity with the underlying tasks might have affected subjects’ performance and mental workload. The investigation of older subjects in connection to learning and mental workload is particularly relevant and meets the evolving needs and expectations of the demographic change of our society and the challenge of life-long learning. An objective method for continuous mental workload registration can offer a way for understanding procedural learning, enhancing skill acquisition, and identifying possible risks.

To conclude, our study was concerned with the neuronal registration of mental workload as connected to planning, strategy learning, and working memory capacity. The topic is of particular interest because of the importance of these constructs for handling a wide range of situations in our digitized world. Understanding the interrelation among them may contribute to adjust conditions, facilitate learning, enhance planning, and reduce workload in accordance to the cognitive abilities of the individual. To the best of our knowledge, there is no other study that investigated planning and mental workload by means of the EEG. We demonstrated the capability of the DFHM index from the EEG to successfully register mental workload and suggest the DFHM method as a useful tool for further studies. In our future research, we aim at employing the DFHM index for the investigation of mental workload related issues of the modern society.

## Data Availability

The conducted data used to support the findings of this study are restricted by the ethics committee of the Federal Institute for Occupational Safety and Health in order to protect subjects privacy according to data-protection regulations. Data can be made available from the corresponding author upon request and after approval of the legal department for researchers who meet the criteria for access to confidential data.

## References

[1] Sternberg, R. J. & Ben-Zeev, T. *Complex Cognition: The Psychology of Human Thought* (OXFORD UNIV PR, 2001).

[2] Carlin D (2000). Planning impairments in frontal lobe dementia and frontal lobe lesion patients. Neuropsychol..

[3] Goel V, Grafman J (1995). Are the frontal lobes implicated in “planning” functions? interpreting data from the tower of hanoi. Neuropsychol..

[4] Goela V, Pullara D, Grafman J (2001). A computational model of frontal lobe dysfunction: working memory and the tower of hanoi task. Cognitive Science.

[5] Cowan N (2013). Working memory underpins cognitive development, learning, and education. Educational Psychology Review.

[6] Brouwer AM (2012). Estimating workload using EEG spectral power and ERPs in the n-back task. Journal of Neural Engineering.

[7] Ke Y (2014). An EEG-based mental workload estimator trained on working memory task can work well under simulated multi-attribute task. Frontiers in Human Neuroscience.

[8] Eggemeier, F., Wilson, G. F., Kramer, A. F. & Damos, D. L. *Multiple-task performance*, chap. Workload assessment in multi-task environments, 207–216 (Taylor & Francis, 1991).

[9] Kahneman, D. *Attention and Effort* (Prentice-Hall, Englewood Cliffs, 1973).

[10] Wickens CD (2002). Multiple resources and performance prediction. Theoretical Issues in Ergonomics Science.

[11] Xie B, Salvendy G (2000). Review and reappraisal of modelling and predicting mental workload in single- and multi-task environments. Work & Stress.

[12] Zoer, I., Ruitenburg, M. M., Botje, D., Frings-Dresen, M. H. W. & Sluiter, J. K. The associations between psychosocial workload and mental health complaints in different age groups. *Ergonomics* **54**, 943–952, 10.1080/00140139.2011.606920 PMID: 21973005 (2011). 10.1080/00140139.2011.606920 21973005

[13] Klonowicz, T. Mental workload and health: A latent threat. *International Journal of Occupational Safety and Ergonomics* **1**, 130–135, 10.1080/10803548.1995.11076309 PMID: 10603543 (1995). 10.1080/10803548.1995.11076309 10603543

[14] Kompier, M. A. J. & Kristensen, T. S. Organisational work stress interventions in a theoretical, methodological and practical context. In Dunham, J. (ed.) *Stress in the Workplace: Past, Present and Future*, 164–190 (Whurr Publishers, London, 2001).

[15] Landsbergis PA, Cahill J, Schnall P (2003). The changing organisation of work and the safety and health of working people: A commentary. Journal of Occupational Environmental Medicine.

[16] NIOSH, N. The changing organization of work and the safety and health of working people. Tech. Rep. 2002–116, National Institute for Occupational Safety and Health (NIOSH) (2002).

[17] Parasuraman R, Molloy R, Singh IL (1993). Performance consequences of automation induced complacency. International Journal of Aviation Psychology.

[18] Sträter, O. Warum passieren menschliche fehler und was kann man dagegen tun? In *Forum Prävention* (AUVA - Allgemeine Unfallversicherungsanstalt, Wien, 2001).

[19] Lehto J (1996). Are executive function tests dependent on working memory capacity?. The Quarterly Journal of Experimental Psychology Section A.

[20] Colom R, Rubio VJ, Shih PC, Santacreu J (2006). Fluid intelligence, working memory and executive functioning. Psicothema.

[21] Miyake A, Friedman NP, Rettinger DA, Shah P, Hegarty M (2001). How are visuospatial working memory, executive functioning, and spatial abilities related? a latent-variable analysis. Journal of Experimental Psychology: General.

[22] Numminen H, Lehto JE, Ruoppila I (2001). Tower of hanoi and working memory in adult persons with intellectual disability. Research in Developmental Disabilities.

[23] Zook NA, Davalos DB, DeLosh EL, Davis HP (2004). Working memory, inhibition, and fluid intelligence as predictors of performance on tower of hanoi and london tasks. Brain and Cognition.

[24] Chan RCK, Wang YN, Cao XY, Chen EYH (2010). Contribution of working memory components to the performance of the tower of hanoi in schizophrenia. East Asian archives of psychiatry: official journal of the Hong Kong College of Psychiatrists = Dong Ya jing shen ke xue zhi: Xianggang jing shen ke yi xue yuan qi kan.

[25] Handley SJ, Capon A, Copp C, Harper C (2002). Conditional reasoning and the tower of hanoi: the role of spatial and verbal working memory. British journal of psychology (London, England: 1953).

[26] Ávila RT (2015). Working memory and cognitive flexibility mediates visuoconstructional abilities in older adults with heterogeneous cognitive ability. Journal of the International Neuropsychological Society.

[27] Diamond A (2013). Executive functions. Annual Review of Psychology.

[28] Miyake, A. *et al*. The unity and diversity of executive functions and their contributions to complex “frontal lobe” tasks: A latent variable analysis. *Cognitive Psychology* **41**, 49–100, 10.1006/cogp.1999.0734 Last accessed on 2014-03-17 (2000). 10.1006/cogp.1999.0734 10945922

[29] Schiff R, Vakil E (2015). Age differences in cognitive skill learning, retention and transfer: The case of the tower of hanoi puzzle. Learning and Individual Differences.

[30] Beaunieux H (2006). Which processes are involved in cognitive procedural learning?. Memory.

[31] Hulme, C. *Working Memory and Severe Learning Difficulties (PLE: Memory)* (Psychology Press, 2014).

[32] Brandenburg J (2014). Working memory in children with learning disabilities in reading versus spelling. Journal of Learning Disabilities.

[33] Alloway TP (2009). Working memory, but not IQ, predicts subsequent learning in children with learning difficulties. European Journal of Psychological Assessment.

[34] Wagner AD (1999). Working memory contributions to human learning and remembering. Neuron.

[35] Swanson HL (1993). Working memory in learning disability subgroups. Journal of Experimental Child Psychology.

[36] Woltz DJ (1988). An investigation of the role of working memory in procedural skill acquisition. Journal of Experimental Psychology: General.

[37] Baddeley, A. D. & Hitch, G. Working memory. In *Psychology of Learning and Motivation*, 47–89, 10.1016/s0079-7421(08)60452-1 (Elsevier, 1974).

[38] Karni A, Sagi D (1993). The time course of learning a visual skill. Nature.

[39] Callan DE (2003). Learning-induced neural plasticity associated with improved identification performance after training of a difficult second-language phonetic contrast. NeuroImage.

[40] Stickgold R, Walker M (2005). Memory consolidation and reconsolidation: what is the role of sleep?. Trends in Neurosciences.

[41] Yerkes, R. M. & Dodson, J. D. The relation of strength of stimulus to rapidity of habit-formation. *Journal of Comparative Neurology and Psychology* **18**, 459–482 Last accessed on 2011-11-03 (1908).

[42] de Waard, D. *The measurement of drivers’ mental workload*. Ph.D. thesis, University of Groningen, Traffic Research Centre, Haren, Netherlands (1996).

[43] Hardy DJ, Wright MJ (2018). Assessing workload in neuropsychology: An illustration with the tower of hanoi test. Journal of Clinical and Experimental Neuropsychology.

[44] Hart, S. G. & Staveland, L. E. Development of the NASA TLX: results of empirical and theoretical research. In Hancock, P. & Meshkati, N. (eds.) *Human Mental Workload*, 139–183 (North Holland, Amsterdam., 1988).

[45] Klimesch W (1999). EEG alpha and theta oscillations reflect cognitive and memory performance: a review and analysis. Brain Research Reviews.

[46] Gevins A (1998). Monitoring working memory load during computer-based tasks with EEG pattern recognition methods. Human Factors: The Journal of the Human Factors and Ergonomics Society.

[47] Gevins, A. & Smith, M. E. Neurophysiological measures of working memory and individual differences in cognitive ability and cognitive style. *Cerebral Cortex* **10**, 829–839, 10.1093/cercor/10.9.829 Last accessed on 2014-02-18 (2000). 10.1093/cercor/10.9.829 10982744

[48] Pfurtscheller G (1997). EEG event-related desynchronization (ERD) and synchronization (ERS). Electroencephalography and Clinical Neurophysiology.

[49] Sterman, M. B. & Mann, C. A. Concepts and applications of EEG analysis in aviation performance evaluation. *Biological Psychology* **40**, 115–130, 10.1016/0301-0511(95)05101-5 EEG in Basic and Applied Settings (1995). 10.1016/0301-0511(95)05101-5 7647174

[50] Borghini, G., Astolfi, L., Vecchiato, G., Mattia, D. & Babiloni, F. Measuring neurophysiological signals in aircraft pilots and car drivers for the assessment of mental workload, fatigue and drowsiness. *Neuroscience & Biobehavioral Reviews* **44**, 58–75, 10.1016/j.neubiorev.2012.10.003 Applied Neuroscience: Models, methods, theories, reviews. A Society of Applied Neuroscience (SAN) special issue (2014). 10.1016/j.neubiorev.2012.10.003 23116991

[51] Baldwin, C. L. & Penaranda, B. N. Adaptive training using an artificial neural network and EEG metrics for within- and cross-task workload classification. *NeuroImage* **59**, 48–56, 10.1016/j.neuroimage.2011.07.047 Neuroergonomics: The human brain in action and at work (2012). 10.1016/j.neuroimage.2011.07.047 21835243

[52] Kohlmorgen, J. *et al*. Improving human performance in a real operating environment through real-time mental workload detection. In Dornhege, G., del R. Millán, J., Hinterberger, T., McFarland, D. & Müller, K. (eds.) *Towards Brain-Computer Interfacing*, 409–422 (MIT Press, Cambridge, 2007).

[53] Lin C-T (2006). Adaptive EEG-based alertness estimation system by using ICA-based fuzzy neural networks. IEEE Transactions on Circuits and Systems I: Regular Papers.

[54] Penaranda BN, Baldwin CL (2012). Temporal factors of EEG and artificial neural network classifiers of mental workload. Proceedings of the Human Factors and Ergonomics Society Annual Meeting.

[55] Wilson, G. F. & Russell, C. A. Real-time assessment of mental workload using psychophysiological measures and artificial neural networks. *Human Factors* **45**, 635–643 Last accessed on 2014-02-18 (2003). 10.1518/hfes.45.4.635.27088 15055460

[56] Gardony AL, Eddy MD, Brunyé TT, Taylor HA (2017). Cognitive strategies in the mental rotation task revealed by EEG spectral power. Brain and Cognition.

[57] Puma S, Matton N, Paubel P-V, Raufaste É, El-Yagoubi R (2018). Using theta and alpha band power to assess cognitive workload in multitasking environments. International Journal of Psychophysiology.

[58] Radüntz T, Scouten J, Hochmuth O, Meffert B (2017). Automated EEG artifact elimination by applying machine learning algorithms to ICA-based features. Journal of Neural Engineering.

[59] Mognon A, Jovicich J, Bruzzone L, Buiatti M (2011). ADJUST: An automatic EEG artifact detector based on the joint use of spatial and temporal features. Psychophysiology.

[60] Radüntz T (2017). Dual frequency head maps: A new method for indexing mental workload continuously during execution of cognitive tasks. Frontiers in Physiology.

[61] Unsworth, N., Heitz, R. P., Schrock, J. C. & Engle, R. W. An automated version of the operation span task. *Behavior Research Methods* **37**, 498–505, 10.3758/BF03192720 Last accessed on 2014-04-02 (2005). 10.3758/bf03192720 16405146

[62] Makeig, S., Bell, A. J., Jung, T.-P. & Sejnowski, T. J. Independent component analysis of electroencephalographic data. In Touretzky, D. S., Mozer, M. C. & Hasselmo, M. E. (eds.) *Advances in Neural Information Processing Systems 8*, 145–151 (MIT Press, 1996).

[63] Hjorth B (1975). An on-line transformation of EEG scalp potentials into orthogonal source derivations. Electroencephalography and Clinical Neurophysiology.

[64] Amin, H. U., Malik, A. S., Badruddin, N. & Chooi, W.-T. Brain behavior in learning and memory recall process: A high-resolution EEG analysis. In *IFMBE Proceedings*, 683–686, 10.1007/978-3-319-02913-9_174 (Springer International Publishing, 2014).

[65] Huang HJ, Kram R, Ahmed AA (2012). Reduction of metabolic cost during motor learning of arm reaching dynamics. Journal of Neuroscience.

